# Indicators of increased ER stress and UPR in aged D2-mdx and human dystrophic skeletal muscles

**DOI:** 10.3389/fphys.2023.1152576

**Published:** 2023-04-25

**Authors:** Swathy Krishna, Hannah R. Spaulding, James E. Koltes, John C. Quindry, Rudy J. Valentine, Joshua T. Selsby

**Affiliations:** ^1^ Department of Animal Science, Iowa State University, Ames, IA, United States; ^2^ School of Integrative Physiology and Athletic Training, University of Montana, Missoula, MT, United States; ^3^ Department of Kinesiology, Iowa State University, Ames, IA, United States

**Keywords:** DMD, mdx, dystrophin, unfolded protein response, transcription factor

## Abstract

Duchenne muscular dystrophy (DMD) is a progressive muscle disease that results in muscle wasting, wheelchair dependence, and eventual death due to cardiac and respiratory complications. In addition to muscle fragility, dystrophin deficiency also results in multiple secondary dysfunctions, which may lead to the accumulation of unfolded proteins causing endoplasmic reticulum (ER) stress and the unfolded protein response (UPR). The purpose of this investigation was to understand how ER stress and the UPR are modified in muscle from D2-mdx mice, an emerging DMD model, and from humans with DMD. We hypothesized that markers of ER stress and the UPR are upregulated in D2-mdx and human dystrophic muscles compared to their healthy counterparts. Immunoblotting in diaphragms from 11-month-old D2-mdx and DBA mice indicated increased ER stress and UPR in dystrophic diaphragms compared to healthy, including increased relative abundance of ER stress chaperone CHOP, canonical ER stress transducers ATF6 and pIRE1α S724, and transcription factors that regulate the UPR such as ATF4, XBP1s, and peIF2α S51. The publicly available Affymetrix dataset (GSE38417) was used to analyze the expression of ER stress and UPR-related transcripts and processes. Fifty-eight upregulated genes related to ER stress and the UPR in human dystrophic muscles suggest pathway activation. Further, based on analyses using iRegulon, putative transcription factors that regulate this upregulation profile were identified, including ATF6, XBP1, ATF4, CREB3L2, and EIF2AK3. This study adds to and extends the emerging knowledge of ER stress and the UPR in dystrophin deficiency and identifies transcriptional regulators that may be responsible for these changes and be of therapeutic interest.

## Introduction

Duchenne muscular dystrophy (DMD) is a fatal muscle disease that affects approximately 1 in 5,000–6,000 boys born worldwide (Mendell et al., 2012; Mah et al., 2014; Ryder et al., 2017; Duan et al., 2021). This disease is caused by the absence of a functional dystrophin protein, a structural component of the dystrophin-glycoprotein complex (DGC), present on the sarcolemmal membrane (Hoffman et al., 1987; Koenig et al., 1987). The absence of dystrophin leads to contraction-induced muscle injuries, fatty and fibrotic infiltration, and muscle wasting (Petrof et al., 1993; Ehmsen et al., 2002; Morris et al., 2010). Dystrophin deficiency also causes a host of cellular dysfunctions including, but not limited to, inflammation, calcium mishandling, impaired autophagy, and oxidative stress (Chen et al., 2005; Selsby et al., 2010; Selsby, 2011; Selsby et al., 2012; Moorwood and Barton, 2014; Spaulding et al., 2018).

The extent to which independent and interdependent modes of cellular dysfunction contribute to DMD pathology remain an active area of scientific inquiry. For instance, the pathophysiological changes associated with DMD may result in perturbations of cellular homeostasis, causing an accumulation of misfolded or unfolded proteins and subsequent ER stress (Moorwood and Barton, 2014; Hulmi et al., 2016; Pauly et al., 2017). In response to these alterations, the unfolded protein response (UPR) is activated in muscle cells. In general, the UPR is thought to be activated primarily through the translocation of three transmembrane sensors: PERK, IRE1α, and ATF6 (Walter and Ron, 2011; Adams et al., 2019; Afroze and Kumar, 2019; Hetz et al., 2020; Gallot and Bohnert, 2021). The most common restorative mechanisms of cellular homeostasis by the UPR are through translational repression to regulate the accumulation of misfolded proteins and through the production of protein chaperones, which facilitate protein folding (Adams et al., 2019; Afroze and Kumar, 2019; Hetz et al., 2020; Gallot and Bohnert, 2021). ER stress may also stimulate proteolysis via activation of the calpain and proteasome systems (Afroze and Kumar, 2019), which may decrease accumulation of misfolded proteins via degradation. This process may be complicated; however, via decreased autophagy in dystrophic muscle (De Palma et al., 2012; Spitali et al., 2013; Spaulding et al., 2018; Krishna et al., 2021), which ostensibly limits removal of protein aggregates.

PERK, IRE1α, and ATF6 are the most studied ER stress transducers that initiate the UPR. Upon accumulation of misfolded proteins in the ER lumen, the chaperone BiP/GRP78/HSPA5 is dissociated from PERK, IRE1α, and ATF6, allowing their activation through phosphorylation and subsequent initiation of the UPR (Walter and Ron, 2011; Hetz, 2012; Afroze and Kumar, 2019). Activated PERK phosphorylates eIF2α at S51, which inhibits global translation, but selectively upregulates ATF4 and ER stress chaperones such as CHOP and BiP/GRP78/HSPA5 (Walter and Ron, 2011; Hetz, 2012; Afroze and Kumar, 2019). eIF2α can also be phosphorylated by PKR/EIF2AK2 as well as GCN2 and HRI, among other kinases (Clemens, 2001). IRE1α-mediated activation of the UPR is initiated by dimerization and phosphorylation of IRE1α. This facilitates splicing of XBP1, forming XBP1s, which promotes transcription (along with translocated and cleaved ATF6) of ER stress chaperones and activation of ERAD (ER-associated degradation) (Walter and Ron, 2011; Hetz, 2012; Afroze and Kumar, 2019). Prolonged ER stress and UPR can also lead to inflammatory signaling and apoptosis. Inflammatory signaling is activated through JNK-AP1 by IRE1α and through NFκB by PERK and IRE1α (Walter and Ron, 2011; Hetz, 2012). Skeletal muscle has an extensive ER; however, a detailed understanding of the role of ER stress in skeletal muscle, particularly under pathophysiological conditions such as DMD, is lacking.

DMD is frequently modeled by the mdx mouse, which has a relatively mild disease phenotype that largely fails to accurately recapitulate progressive degeneration in skeletal muscle as observed in dystrophin-deficient human muscle, with the notable exception of the diaphragm (Stedman et al., 1991; Grounds et al., 2008). In the mdx model, lifespan is only slightly shortened, and heart function is largely preserved (Chamberlain et al., 2007; Grounds et al., 2008; Yucel et al., 2018). Despite this, the mdx mouse model has been instrumental for the determination of disease mechanisms and cellular dysfunction and as an early screen of therapeutic interventions. Consistent with the aforementioned understanding of cellular dysfunction, a limited body of work suggests dystrophin deficiency causes ER stress in muscles from mdx mice and those with DMD (Moorwood and Barton, 2014; Hulmi et al., 2016; Pauly et al., 2017). Given the relatively mild phenotype in the mdx mouse model, the mdx mutation was backcrossed to a DBA line, resulting in a more severe model (D2-mdx), which better recapitulates human disease pathology (Coley et al., 2016; Putten et al., 2019; Spaulding et al., 2019; Hammers et al., 2020). The extent to which ER stress may be stimulated in this emerging murine model is unknown and in muscles from boys/men with DMD has only been briefly considered (Moorwood and Barton, 2014). Therefore, the purpose of this investigation was to determine the extent to which ER stress is modified in dystrophin-deficient skeletal muscle in an emerging mouse model and in boys/men with DMD. This improved understanding will be beneficial in the development of therapeutics aimed at correcting secondary dysfunctions associated with DMD. We hypothesized that markers of ER stress and UPR would be upregulated in 11-month-old D2-mdx diaphragm and human dystrophic muscles.

## Methods

### Animal treatments

Animal treatments were approved by the Institutional Animal Care and Use Committees at the University of Montana and the University of Florida. Detailed methods and data from limb muscle (Spaulding et al., 2019), and diaphragm (Spaulding et al., 2020; Krishna et al., 2021), from these animals have been previously published. In brief, 11-month-old DBA (*n* = 7) or D2-mdx (*n* = 7) male mice were anesthetized to a surgical level, and diaphragms were collected, and then the mice were euthanized by exsanguination. The collected muscles were frozen in liquid nitrogen for further analyses. Previously reported functional analyses from these diaphragms indicated reduced specific force and histopathological analyses indicated increased fibrotic area and reduced contractile area in diaphragms from D2-mdx compared to diaphragms from DBA mice (Spaulding et al., 2020). Given these previously established functional, histological, and biochemical alterations, the diaphragm was selected for experiments described herein.

### Protein extraction and Western blotting in D2-mdx

Diaphragms were prepared for Western blotting as previously described (Krishna et al., 2021). In brief, protein was extracted in whole muscle buffer (10 mM sodium phosphate, 2% SDS, pH 7.0) and the concentration was determined using BCA (Thermo Fisher Pierce^TM^ BCA protein assay, #23225). Protein was diluted to a concentration of 4 μg/μL and precisely 40 µg of protein was loaded into each lane. Protein was separated by molecular mass and then transferred to a nitrocellulose membrane. Equal loading was confirmed by quantification of total lane optical density following a Ponceau S stain and objective measurement using AzureSpot software (Azure Biosystems, Version 2.0.062). The stain was removed, membranes were blocked in 5% milk in Tris-buffered saline with 0.1% Tween 20 (TBST), and membranes were incubated with primary and secondary antibodies (Table 1). The membranes were then exposed to enhanced chemiluminescence blotting substrate (ECL, BioRad Clarity^TM^) for 5 min, imaged using Azure^TM^ C600, and quantified using AzureSpot software using automated band detection.

**TABLE 1:** Primary and secondary antibodies used for Western blotting.

Antibody	Product id	Company	Host	Primary	Secondary
AP1	A5968	Sigma aldrich	Rabbit	1:1000 TBST	1:2000 TBST
ATF4	97038 S	Cell signaling	Mouse	1:1000 TBST	1:2000 TBST
ATF6	65880 S	Cell signaling	Rabbit	1:1000 TBST	1:2000 TBST
BiP	3,177	Cell signaling	Rabbit	1:1000 TBST	1:2000 TBST
CHOP	2,895	Cell signaling	Mouse	1:1000 TBST	1:2000 TBST
eIF2α	9722 S	Cell signaling	Rabbit	1:1000 TBST	1:2000 TBST
IRE1α	3,294	Cell signaling	Rabbit	1:1000 TBST	1:2000 TBST
IκBα	4,812	Cell signaling	Rabbit	1:1000 TBST	1:2000 TBST
NFκB	8,242	Cell signaling	Rabbit	1:1000 TBST	1:2000 TBST
peIF2α S51	3,398	Cell signaling	Rabbit	1:1000 TBST	1:2000 TBST
pIRE1α S724	Ab48187	Abcam	Rabbit	1:1,000 2.5% milk	1:1000 TBST
PKR	SC-6282	Santa cruz biotechnology	Rabbit	1:1000 TBST	1:2000 TBST
pNFκB S536	3,033	Cell signaling	Rabbit	1:1000 TBST	1:2000 TBST
pPERK T980	3,179	Cell signaling	Rabbit	1:3,000 2.5% milk	1:3,000 5% milk
pPKR T446	Ab32036	Abcam	Rabbit	1:1000 TBST	1:2000 TBST
XBP1s	40435 S	Cell signaling	Rabbit	1:1000 TBST	1:2000 TBST

### Human affymetrix chip data analysis

To evaluate ER stress in human dystrophic muscle, the publicly available Affymetrix microarray dataset accession ID: GSE38417 (alternate ID: EGEOD-38417) (Dorsey and Ward, 2016) was analyzed using the Bioconductor package, Limma (Ritchie et al., 2015) in R (Team, 2021). The microarray data were generated from muscle biopsy samples from boys with (*n* = 12; range: 0.92–8 years; mean: 3.75 ± 2.25 years)) or without (*n* = 6; age unknown) DMD using Human U1133 2.0 arrays. Gene Ontology (GO) analysis and network constructions were performed using ClueGO in Cytoscape version 8.0 (Bindea et al., 2009). String database (Szklarczyk et al., 2021) was used to identify the interactions between the genes of interest. Specifically, the experimentally determined and predicted interactions, based on curated databases and text mining, between the genes were mapped into the network. The transcriptional regulators of the genes of interest were identified using the iRegulon plugin (Janky et al., 2014) in Cytoscape.

### Statistics

For the statistical analysis of western blots, an unpaired *t*-test was performed in GraphPad Prism version 8.0.0. Significance was established at *p* < 0.05. For differential expression in the microarray, the Limma package in R was used (Ritchie et al., 2015), and significance for differential expression was established at a log_2_ fold change (FC) > 0.2 and false-discovery rate (FDR) < 0.1 [applying Benjamini and Hochberg (BH) correction (Benjamini and Hochberg, 1995)] for all analyses. For the ontology analyses, an FDR < 0.1 was applied.

## Results

### ER stress in D2-mdx mice

These mice and tissues have been used to support previous investigations. Through this previous work, we have established that diaphragms from these D2-mdx mice have histopathological injury and impaired specific tension compared to DBA controls (Spaulding et al., 2020) as well as impaired autophagy (Krishna et al., 2021). We have also established that limb muscle from these D2-mdx mice have smaller mass (absolute and relative), decreased function, and increased histopathological injury compared to DBA mice (Spaulding et al., 2020) as well as alterations in autophagy (Krishna et al., 2021).

**FIGURE 1:**
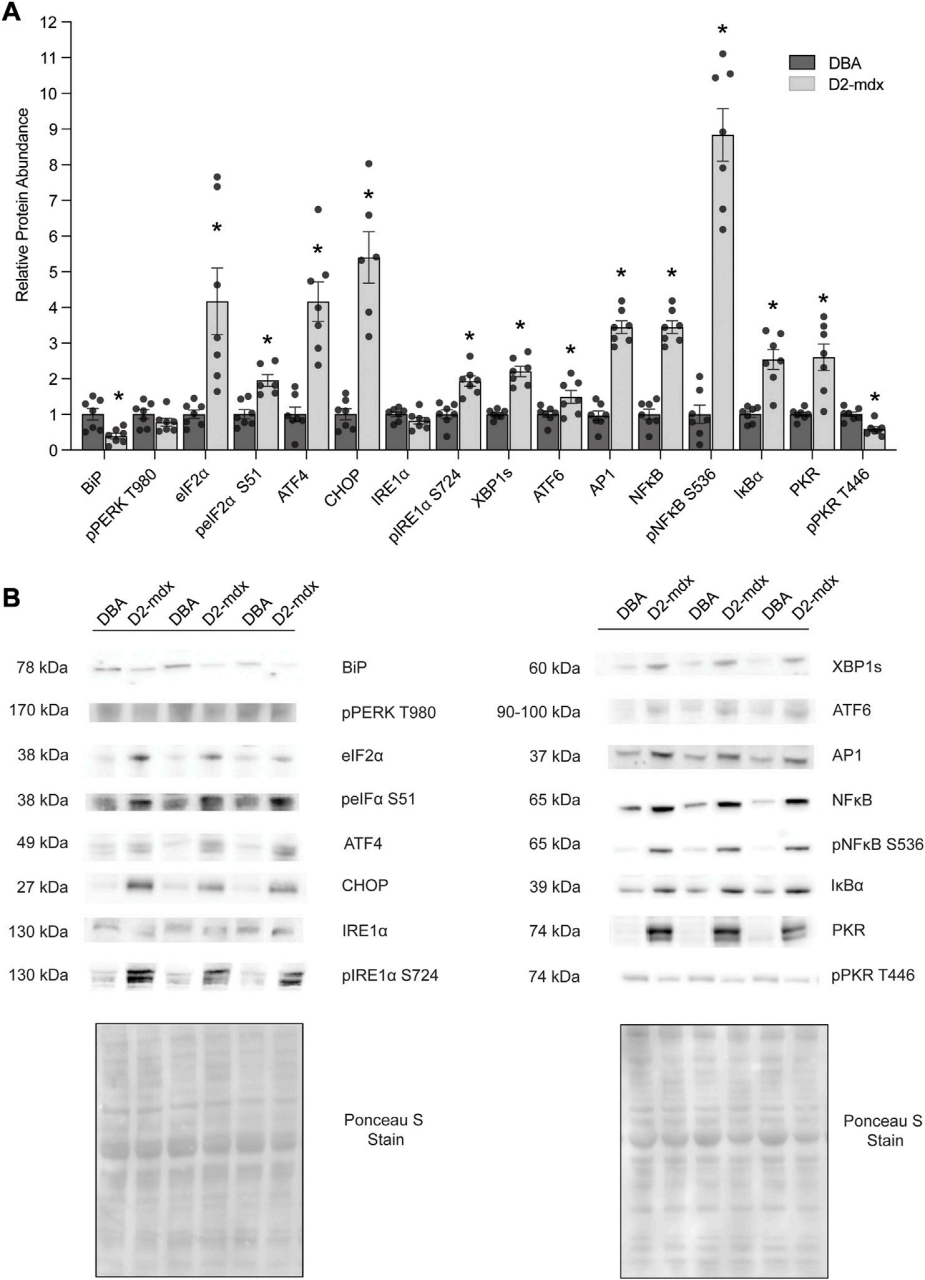
Increased abundance of markers of ER stress and the UPR in D2-mdx mice (*n* = 7) compared to DBA (*n* = 7). **(A)** Increased relative abundance except for BiP/GRP78/HSPA5 and pPKR T446 (decreased), IRE1α (similar), and pPERK T980 (similar). **(B)** Representative Western blot and Ponceau S stain images. Unpaired *t*-test used for statistical analysis. Significance established at *p* < 0.05.

In 11-month-old D2-mdx diaphragm, the ER chaperone BiP/GRP78/HSPA5 was decreased by approximately 60% (*p* = 0.0054) compared to DBA (Figure 1). Similarly, pPERK T980 (PERK phosphorylated at T980) was numerically, albeit non-significantly, decreased (*p* = 0.0560). Dystrophin deficiency increased eIF2α approximately 4-fold (*p* = 0.0056) and peIF2α S51 was increased approximately 2-fold (*p* = 0.0009) compared to DBA. ATF4 was increased approximately 4-fold (*p* = 0.0002) and CHOP was increased approximately 6.2-fold (*p* < 0.0001) in D2-mdx compared to DBA. While IRE1α (*p* = 0.1572) was similar between groups, activated pIRE1α S724 was increased approximately 2-fold (*p* = 0.0003) compared to DBA. Further, the IRE1α-mediated spliced XBP1 (XBP1s) was increased by 2.2-fold (*p* < 0.0001), and ATF6 by approximately 1.5-fold (*p* = 0.0298) in D2-mdx compared to DBA.

In addition to chronic muscle injury, prolonged ER stress and UPR cause activation of inflammatory signaling. In D2-mdx, relative abundance of AP1 was increased by approximately 2.5-fold compared to DBA. There was an increased relative abundance of NFκB (by approximately 3.5-fold, *p* < 0.0001) and pNFκB S536 (by 8.8-fold, *p* < 0.0001) in D2-mdx, despite increased IκBα (an inhibitor of NFκB activity; 2.5-fold, *p* = 0.0002), compared to healthy muscles. Interestingly pPKR T446, which regulates ER stress and inflammatory pathways, was decreased by approximately 40% (*p* = 0.0010), although total PKR/EIF2AK2 was increased by 2.6-fold (*p* = 0.0011).

### ER stress in skeletal muscle from boys with DMD

**FIGURE 2:**
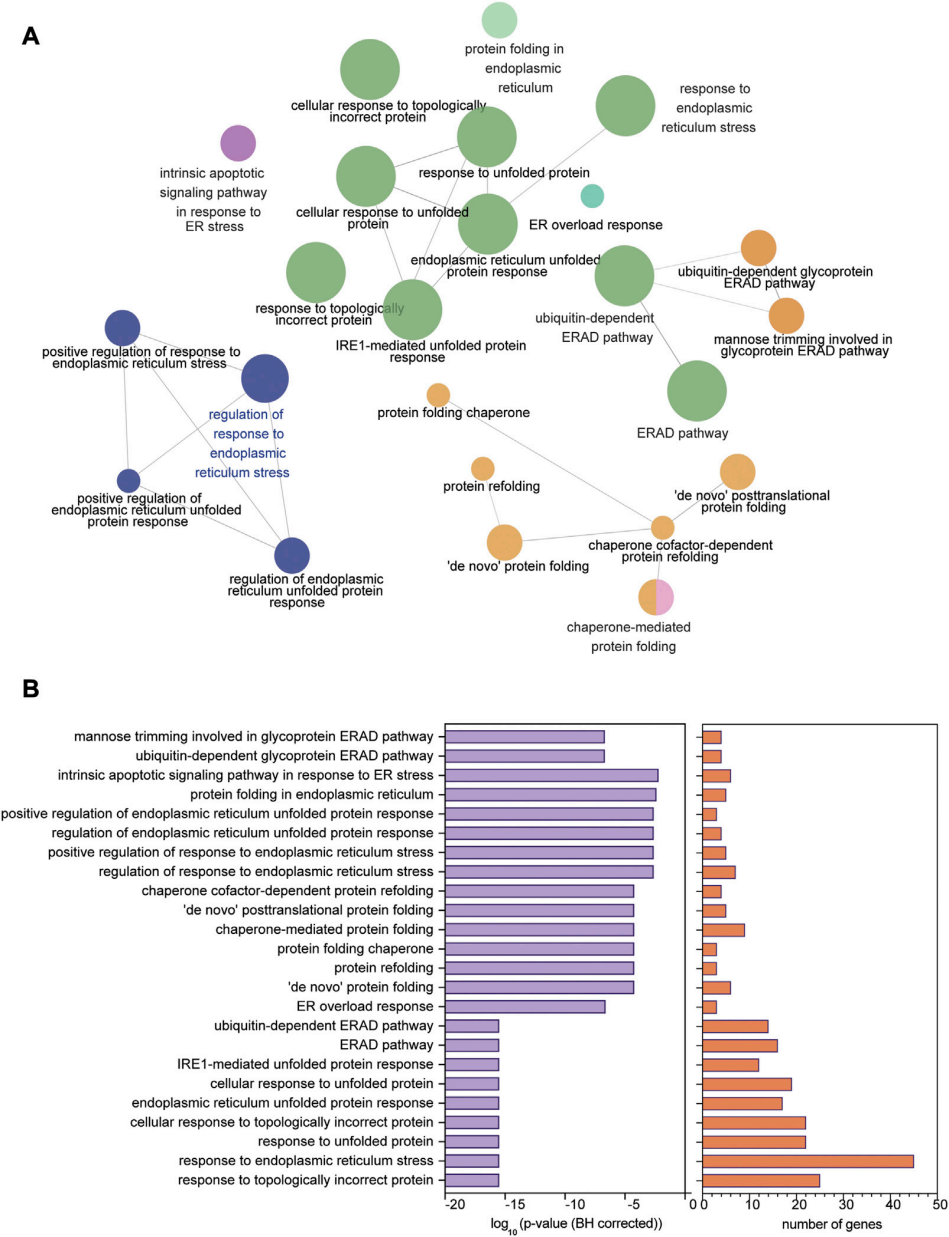
ER stress and UPR-related processes identified in human dystrophic muscle. **(A)** Ontology processes related to ER stress and the UPR analyzed using ClueGO in upregulated genes from the human Affymetrix data chip analysis mapped in Cytoscape. **(B)** *p*-value (BH corrected) and number of genes in each of the 24 processes identified. Significance of enrichment established at FDR < 0.01 (BH correction).

Ontology analyses (for biological processes and cell components) to identify processes related to the ER were performed separately on the upregulated (FDR<0.1 and log_2_ FC > 0.2) and downregulated (FDR<0.1 and log_2_ FC < 0.2) genes. We did not identify enriched ER-related processes using the ontology analysis of the downregulated genes. We identified 553 genes using GO biological processes and 449 genes using GO cellular components related to the ER following ontology analyses on the upregulated genes. When combined, a total of 868 unique genes were used for a refined search to identify specific processes related to ER stress and the UPR. Through this approach, we identified 24 processes comprised of a total of 58 genes (Figures 2A,B; Table 2). The processes identified suggest possible disruptions in protein folding, calcium storage and release, and activation of the UPR and ERAD (Table 2). Of note, among the processes identified, “response to endoplasmic reticulum stress” had the most identified genes (45 genes) (Figure 2B).

**TABLE 2:** Gene ontology (GO) processes identified for the 58 upregulated ER stress- and UPR-related genes from the human Affymetrix chip analysis with p-adjusted (BH) < 0.1. The ontology analyses for all the differentially expressed genes were performed in ClueGO in Cytoscape. No significantly enriched ER stress- and UPR-related processes were identified with downregulated genes.

	GO terms	Process	Genes
1	GO:0035966	response to topologically incorrect protein	[ARFGAP3, ATXN3, CANX, CREB3L2, DNAJB11, DNAJB14, EDEM1, EDEM3, EXTL2, FKBP14, HSP90B1, HSPA13, KDELR3, PDIA5, PIK3R1, PTPN2, SERP1, SERPINH1, SSR1, THBS1, TM7SF3, TMEM33, TPP1, UFL1, UGGT2]
2	GO:0034976	response to ER stress	[ALOX5, ARFGAP3, ATXN3, BID, CANX, CASP4, CAV1, CCDC47, CREB3L2, DNAJB11, DNAJB14, DNAJC10, EDEM1, EDEM3, ERLIN1, ERLIN2, EXTL2, FKBP14, HSP90B1, ITPR1, KDELR3, MAN1A1, MAN1B1, MAP3K5, OS9, PDIA3, PDIA5, PIK3R1, PTPN2, SEC16A, SERP1, SRPX, SSR1, THBS1, TMCO1, TMEM33, TMTC3, TMX1, TP53, TPP1, TRIM25, UBE2J1, UFL1, UFM1, UGGT2]
3	GO:0006986	response to unfolded protein	[ARFGAP3, CANX, CREB3L2, DNAJB11, EDEM1, EDEM3, EXTL2, FKBP14, HSP90B1, HSPA13, KDELR3, PDIA5, PIK3R1, PTPN2, SERP1, SERPINH1, SSR1, THBS1, TM7SF3, TMEM33, TPP1, UFL1]
4	GO:0035967	cellular response to topologically incorrect protein	[ARFGAP3, ATXN3, CANX, CREB3L2, DNAJB11, DNAJB14, EDEM1, EXTL2, FKBP14, HSP90B1, HSPA13, KDELR3, PDIA5, PIK3R1, PTPN2, SERP1, SSR1, TM7SF3, TMEM33, TPP1, UFL1, UGGT2]
5	GO:0030968	ER unfolded protein response	[ARFGAP3, CANX, CREB3L2, DNAJB11, EDEM1, EXTL2, FKBP14, HSP90B1, KDELR3, PDIA5, PIK3R1, PTPN2, SERP1, SSR1, TMEM33, TPP1, UFL1]
6	GO:0034620	cellular response to unfolded protein	[ARFGAP3, CANX, CREB3L2, DNAJB11, EDEM1, EXTL2, FKBP14, HSP90B1, HSPA13, KDELR3, PDIA5, PIK3R1, PTPN2, SERP1, SSR1, TM7SF3, TMEM33, TPP1, UFL1]
7	GO:0036498	IRE1-mediated unfolded protein response	[ARFGAP3, DNAJB11, EDEM1, EXTL2, FKBP14, KDELR3, PDIA5, SERP1, SSR1, TMEM33, TPP1, UFL1]
8	GO:0036503	ERAD pathway	[ATXN3, CAV1, CCDC47, DNAJB14, DNAJC10, EDEM1, EDEM3, ERLIN1, ERLIN2, HSP90B1, MAN1A1, MAN1B1, OS9, TRIM25, UBE2J1, UGGT2]
9	GO:0030433	ubiquitin dependent ERAD pathway	[CAV1, CCDC47, DNAJB14, DNAJC10, EDEM1, EDEM3, ERLIN1, ERLIN2, HSP90B1, MAN1A1, MAN1B1, OS9, TRIM25, UBE2J1]
10	GO:0006983	ER overload response	[BID, CCDC47, TMCO1]
11	GO:0006458	“*de novo*” protein folding	[CD74, DNAJB14, FKBP1B, GAK, HSPA13, SELENOF]
12	GO:0042026	protein refolding	[B2M, FKBP1B, HSPA13]
13	GO:0044183	protein folding chaperone	[CCDC47, CD74, HSPA13]
14	GO:0061077	chaperone-mediated protein folding	[CD74, CRTAP, CSNK2A2, DNAJB14, FKBP1B, GAK, HSPA13, P3H1, PPIB]
15	GO:0051084	“*de novo*” post translational protein folding	[CD74, DNAJB14, GAK, HSPA13, SELENOF]
16	GO:0051085	chaperone cofactor-dependent protein refolding	[CD74, DNAJB14, GAK, HSPA13]
17	GO:1905897	regulation of response to ER stress	[ALOX5, ATXN3, CAV1, PIK3R1, PTPN2, TMEM33, UFL1]
18	GO:1905898	positive regulation of response to ER stress	[ATXN3, CAV1, PIK3R1, PTPN2, TMEM33]
19	GO:1900101	regulation of ER unfolded protein response	[PIK3R1, PTPN2, TMEM33, UFL1]
20	GO:1900103	positive regulation of ER unfolded protein response	[PIK3R1, PTPN2, TMEM33]
21	GO:0034975	protein folding in ER	[CANX, DNAJC10, HSP90B1, PDIA3, VAPA]
22	GO:0070059	intrinsic apoptotic signaling pathway in response to ER stress	[CASP4, DNAJC10, ITPR1, MAP3K5, PTPN2, TP53]
23	GO:0097466	ubiquitin-dependent glycoprotein ERAD pathway	[EDEM1, EDEM3, MAN1A1, MAN1B1]
24	GO:1904382	mannose trimming involved in glycoprotein ERAD pathway	[EDEM1, EDEM3, MAN1A1, MAN1B1]

**FIGURE 3:**
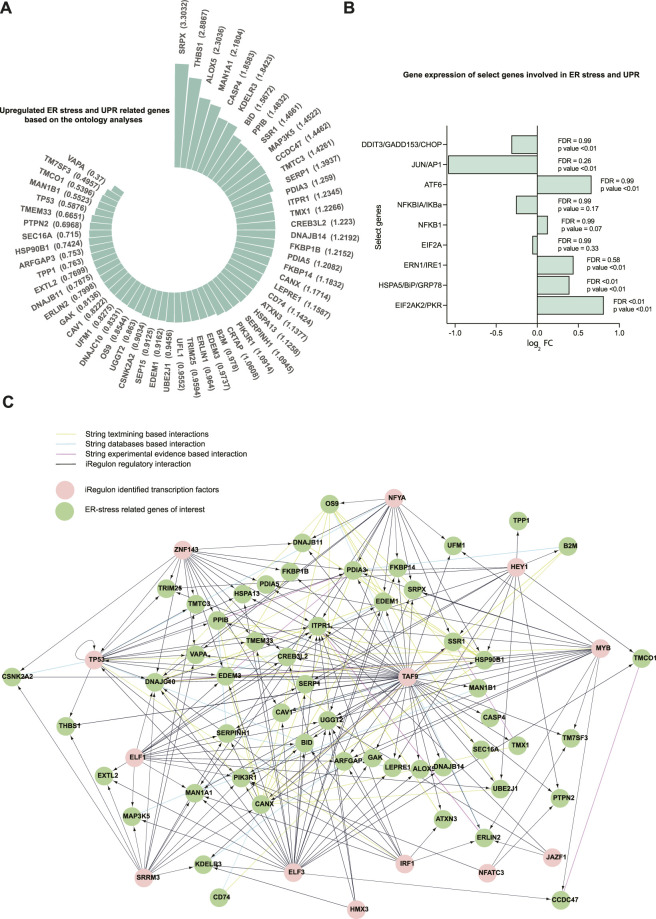
A total of 58 upregulated genes related to ER stress and the UPR were identified in the human Affymetrix chip data analyzed. **(A)** Fold changes (log_2_ FC) for the 58 upregulated ER stress and UPR-related genes (FDR ranged from 2E-09 to 0.03). Differential expression was analyzed using the Limma package in R. The 58 genes were selected from the 24 GO processes that are related to ER stress and the UPR. **(B)** Representation of gene expression of select genes subsetted from the human Affymetrix chip data corresponding to the proteins immunoblotted in DBA and D2-mdx mice. **(C)** The network for the genes of interest and their transcriptional regulators is created using Cytoscape incorporating the String interactions (with a high interaction score of 0.7) and transcription factors identified using iRegulon (NES cutoff = 4). The top 13 transcriptional regulators are used.

The 58 upregulated genes had log_2_ FC ranging from 0.3699 to 3.3031 (Figure 3A), and FDR ranged from 2E-09 to 0.03 (Supplementary Table S1) in dystrophic muscles compared to healthy muscles. Additionally, a subset of the human genes that correspond to the proteins measured in D2-mdx and DBA mice were also detected in the microarray dataset (Figure 3B). Among these genes, only PKR/EIF2AK2 was upregulated considering the stringent cut-off of FDR < 0.1 (log_2_ FC of 0.08, FDR = 0.003, *p* < 0.01), whereas if *p*-value (*p* < 0.05) was considered several additional transcripts were also upregulated.

Next, we identified transcription factors that may contribute to the regulation of the identified 58 genes. Based on sequence analysis and motif searches, we identified 30 putative transcription factors (normalized enrichment score (NES) cut-off 3%) that regulate ER stress and UPR-related genes of interest (Table 3). This analysis identified transcription factors that regulate the majority (86.2%) of the upregulated ER stress and UPR-related genes identified in our analysis. Next, we queried our dataset and discovered that, of these 30 transcription factors, 27 were differentially expressed (FDR<0.1; 14 downregulated and 13 upregulated; Supplementary Table S2). In order to identify the most plausible transcriptional regulatory profile, the top 13 transcriptional regulators (NES cut-off 4.00) and the identified ER-stress-related genes regulated by these transcription factors were mapped into a network (Figure 3C) along with known and predicted interactions identified using String. Of interest, the transcriptional regulators TAF9, NFYA, and TP53 were among the identified String interactions that were experimentally determined (Supplementary Figure S1), providing additional confidence in this approach.

**TABLE 3:** Putative transcription factors with their orthologues and target genes identified based on motif and track searches in iRegulon. Transcription factors with a normalized enrichment score (NES) > 3% and higher area under curve (AUC) are listed. The iRegulon analysis was performed on the 58 upregulated genes from the identified ER stress- and UPR-related processes.

Transcription factors	Target genes	NES	AUC
TAF9, CREB3L1, CREB3, XBP1, ATF6, ATF6B, CREB3L2	SERPINH1, SSR1, LEPRE1, FKBP14, KDELR3, CANX, THBS1, PDIA5, UBE2J1, DNAJC10, TMEM33, HSP90B1, MAN1A1, PPIB, TMX1, CAV1, FKBP1B, CSNK2A2, EDEM3, DNAJB11, BID, EDEM1, ALOX5, SERP1, TM7SF3, UGGT2	8.896	0.1161
CREB3L2	SERPINH1, LEPRE1, FKBP14, KDELR3, PDIA5, MAN1A1, CAV1, ARFGAP3, ALOX5	7.6388	0.1013
ZNF143, TP53	VAPA, TMTC3, EDEM3, ITPR1, DNAJC10, TP53, PDIA3, PIK3R1	5.7753	0.0792
ELF3, ELK1, GABPA, ERF, ELK3, ETV3, ETS1, ETV1, FEV, ERG, ELK4, FLI1, ETV5, ETV4, ETS2, GABPB1, ETV2, ELF1, ETV6, ELF4, ELF5	EDEM3, TMCO1, SERP1, UGGT2, TMEM33, EXTL2, TP53, ITPR1, PIK3R1, LEPRE1, DNAJC10, DNAJB14, MAP3K5, MAN1A1, HSPA13	4.9077	0.0689
CDX1, MYB, NFE2L1, TLX2, HNF1B, HNF1A, PAX4	ERLIN2, PIK3R1, CSNK2A2, ITPR1, UGGT2, CAV1, SRPX, MAN1A1, LEPRE1, DNAJC10	4.8236	0.0679
NFATC3, ZEB1	PTPN2, PIK3R1, TM7SF3	4.5491	0.0646
NFYC, NFYA, NFYB, HNF1B, HNF1A, POLE3	DNAJB11, FKBP14, HSP90B1, VAPA	4.3964	0.0628
SRRM3	MAN1A1, PIK3R1, MAP3K5, EXTL2, CSNK2A2	4.2415	0.061
HEY1	DNAJC10, TMCO1, LEPRE1, SERP1, B2M, UBE2J1, TPP1, TRIM25, PDIA3, SSR1, SERPINH1, HSP90B1, PTPN2	4.2012	0.0857
ELF1, NEUROD1, TAL1	UBE2J1, EDEM1, MAN1A1, PIK3R1, MAP3K5, SERPINH1, EDEM3, UGGT2, DNAJC10, CREB3L2, ITPR1, BID, ARFGAP3	4.1884	0.0604
HMX3, HMX1	ITPR1, CREB3L2, UGGT2, KDELR3	4.1485	0.0599
SPI1	ITPR1, MAP3K5, B2M, BID, EDEM3, PPIB, PTPN2, EDEM1	4.0795	0.0838
IRF1, IRF7, IRF8	MAN1A1, PIK3R1, ATXN3, BID	4.0688	0.059
JAZF1	ERLIN2, ITPR1, UGGT2	4.0556	0.0588
SPIB, SPI1, SPIC, ETV6, ELF5, ELK1, ETV7, ETS1, SIRT6, GABPB1, ELF4, ELF2, ELK4, FLI1, YY1, NFATC3, TCF4, TBP	BID, ALOX5, MAP3K5, HSPA13, DNAJC10, EDEM3, TMCO1, PIK3R1, ITPR1, CD74	3.9626	0.0577
CEBPG, CEBPB, CEBPE, CEBPD, TBP	TMEM33, CCDC47, CSNK2A2, TM7SF3, ERLIN2	3.9427	0.0575
HOXC13, HOXA13, HOXB13, HOXD13, HOXD12, HOXC10, HOXC12, HOXC11, HOXA11, HOXA10, CDX1, OVOL1	TM7SF3, ITPR1, PIK3R1, MAP3K5, CAV1, TMEM33	3.5244	0.0525
EBF1	ARFGAP3, ALOX5, TMX1, EDEM1, PTPN2, CREB3L2, UFM1, MAP3K5	3.4896	0.0745
POLR2A	TP53, B2M, TPP1, TRIM25, EXTL2	3.4463	0.0738
TFAP4, FOXO1, FOXA1	SERPINH1, KDELR3, CREB3L2, PIK3R1, ITPR1	3.3761	0.0507
GFI1,GFI1B	SERPINH1, TMEM33, TMTC3	3.365	0.0506
YY1	HSPA13, CANX, TP53, EDEM1, B2M	3.3413	0.0722
MAX	ATXN3, PDIA5, MAN1B1, ARFGAP3, CCDC47, TMEM33	3.2547	0.0708
TEAD4	THBS1, UGGT2, TMCO1, CAV1, TRIM25	3.2513	0.0708
HNF4A, NR2F1, NR2F2, HNF4G, RXRG, PPARG, RXRB, RXRA, NR2C2	TM7SF3, PIK3R1, CSNK2A2, MAN1A1, UFM1	3.2101	0.0488
HSF1, HSF4	PIK3R1, SERPINH1, CD74, TMEM33	3.0839	0.0473
GATA2, GATA5, ZNF217, GATA6, GATA4, GATA3, GATA1	ITPR1, TM7SF3, SRPX	3.0773	0.0472
MZF1	CANX, PPIB, PDIA3, GAK, MAN1B1, UGGT2, DNAJC10, SERP1, TRIM25, TMTC3, SSR1, LEPRE1, SRPX, PTPN2, OS9, EXTL2, TP53, B2M, VAPA, UBE2J1, CCDC47	3.0197	0.0671
RFX5	B2M, TPP1, CD74	3.0164	0.0671

**FIGURE 4:**
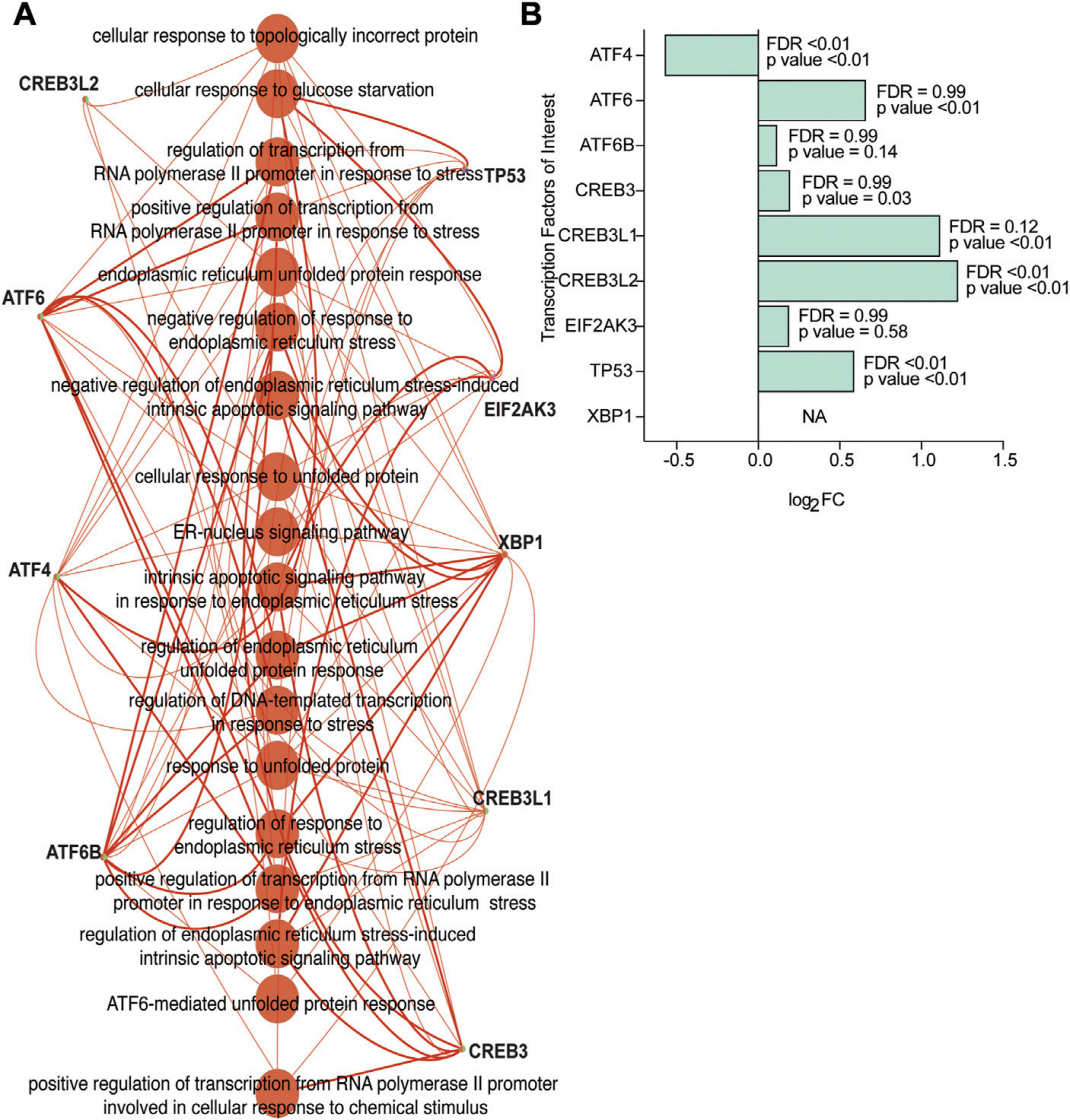
Transcriptional regulators from the human Affymetrix dataset confirmed as linked to ER stress and UPR-related processes. **(A)** The transcriptional regulators CREB3L2, ATF6, ATF4, ATF6B, TP53, EIF2AK3, XBP1, CREB3L1, and CREB3 correspond to ER-stress and UPR-related enriched processes based on gene ontology analysis on all the identified transcription factors, and orthologues (NES>3.00) identified using iRegulon. **(B)** Gene expression levels of the transcription factors of interest (based on a higher NES value and ontology analysis on transcription factors) subsetted from the human Affymetrix chip data analysis. FDR and *p*-values from differential expression using the Limma package are included.

An ontology analysis on the identified transcriptional regulators and their orthologues was performed to determine if they were previously described in association with ER and ER stress. Our analysis confirmed CREB3L2, ATF6, ATF4, ATF6B, TP53, EIF2AK3, XBP1, CREB3L1, and CREB3 (Figure 4A) were associated with ER and ER stress. Additionally, our analysis also suggested that transcription factors TAF9, ELK1, MYB, and NFATC3, which had a high NES value, were also associated with ER and ER stress (Figure 4B).

## Discussion

Dystrophin deficiency causes multiple, deleterious changes to cellular processes and leads to oxidative stress, impaired calcium homeostasis, inflammation, and impaired autophagy. Disruption of cellular homeostasis may also result in accumulation of misfolded proteins and subsequent ER stress, which can lead to the activation of the UPR as well as inflammation and ultimately activation of cell death pathways (Hetz, 2012; Moorwood and Barton, 2014; Pauly et al., 2017; Afroze and Kumar, 2019; Gallot and Bohnert, 2021). The extent to which ER stress and the UPR occur in dystrophic muscle remain poorly understood. The purpose of this study was to better understand ER stress and the UPR in the emerging D2-mdx model and in human dystrophic skeletal muscles. Herein, we provide new evidence for ER stress and the UPR in a murine dystrophinopathy model and in human dystrophic muscle. Additionally, based on sequence and motif analysis from humans, we identified potential transcriptional regulators of ER stress and the UPR in dystrophic skeletal muscle.

The D2-mdx model is an emerging mouse model of DMD and may better recapitulate disease pathology and progression than other commonly used dystrophin-deficient mouse models (Putten et al., 2019; Spaulding et al., 2019; Hammers et al., 2020; Spaulding et al., 2020). The extent to which ER stress and the UPR are modified in the D2-mdx model is unclear, although ER stress and the UPR were previously described in the well-studied DMD model, the mdx mouse (Moorwood and Barton, 2014; Hulmi et al., 2016). Herein, we report that markers of ER stress and the UPR were upregulated in diaphragm from 11-month-old D2-mdx mice, in good agreement with findings in dystrophic muscle from mdx mice (Moorwood and Barton, 2014; Hulmi et al., 2016; Pauly et al., 2017). In dystrophic diaphragms from mdx mice, markers of ER stress and the UPR were increased, but these processes may be mediated in part by age or disease progression. When viewed within this context, our data expand upon existing knowledge that indicates increased ER stress and the UPR have a more severe phenotype in the advanced disease model (D2-mdx mice) than in the less severe mdx model (Moorwood and Barton, 2014; Pauly et al., 2017). Moreover, and contrary to findings from studies reporting increased BiP/GRP78/HSPA5 in skeletal muscles from mdx mice (Moorwood and Barton, 2014; Pauly et al., 2017), we discovered BiP/GRP78/HSPA5 was decreased in diaphragms from D2-mdx mice likely due to age, genetic background, or advanced disease severity in animals used herein. Reduced expression of some ER chaperones with aging has been reported in skeletal muscle, suggestive of decreased ability to cope with ER stress (Ogata et al., 2009; Deldicque, 2013; O'Leary et al., 2013). These findings of increased ER stress and UPR due to the accumulation of misfolded proteins are complementary to our previous findings of an accumulation of autophagosomes in these dystrophic diaphragms (Krishna et al., 2021). It seems reasonable to suggest that an accumulation of damaged proteins and protein aggregates due to blunted autophagy, despite increased activation of calpain and proteasome systems (Selsby et al., 2010; Selsby et al., 2012; Hollinger and Selsby, 2013) is sufficient to cause ER stress and the UPR.

As a consequence of the UPR, eIF2α may be phosphorylated (S51) by a variety of kinases including PKR/EIF2AK2 and PERK, which antagonizes translation (Clemens, 2001). This flexibility supports our finding of increased peIF2α S51 despite similar pPERK T986 and decreased pPKR T446. Furthermore, PKR/EIF2AK2 regulates additional cell signaling and stress-related responses, including inflammatory signaling in response to ER stress (Gal-Ben-Ari et al., 2018). As increased activation of NFκB, as reported herein, is a hallmark of dystrophic muscle, these data raise the possibility that ER stress may contribute to this outcome. The associated elevation of the endogenous NFκB inhibitor, IκBα, may represent an attempt to limit inflammatory signaling despite the overwhelming pro-inflammatory muscle environment. Pharmacologic inhibition of ER stress in dystrophic muscle improved sarcoplasmic reticulum (SR)/ER-mitochondria interaction, calcium homeostasis, and muscle contractility (Pauly et al., 2017), suggesting increased ER stress, or at least damage or dysfunction of the ER, is part of the disease sequala. Moreover, that disease severity was attenuated in a dystrophic mouse model following the knockout of caspase-12, an ER-specific caspase activated in muscles from mdx mice and humans with DMD (Moorwood and Barton, 2014), further supports this notion. Collectively, these studies raise the possibility of the therapeutic importance of ER stress- and UPR-related changes in DMD.

Despite a growing body of literature supporting ER stress and the UPR in dystrophic muscle from mouse models, human data is limited, to our knowledge, to increased relative abundance of cleaved caspase-4 and BiP in muscle from boys with DMD (Moorwood and Barton, 2014). Publicly available datasets from human dystrophic biopsies are an important resource to address novel research questions as they do not require new biopsies or experimentation, which may consume limited available tissue. Despite the paucity of human data for comparison, data presented in the present investigation are consistent with this previous report (Moorwood and Barton, 2014) in that processes related to ER stress and the UPR were upregulated. Specifically, within the transcriptomic dataset, we identified 58 upregulated genes related to ER stress and the UPR that were involved in processes associated with the disruption of calcium storage and release, protein folding and secretion, lipid biogenesis, and activation of ERAD, inflammation, and apoptosis in dystrophic muscles.

We also directly compared our protein expression data from D2-mdx mice to our gene expression data to identify commonalities. Although BiP/GRP78/HSPA5 protein abundance was decreased in D2-mdx mice and gene expression was similar in healthy and dystrophic human muscles, gene expression of BiP/GRP78/HSPA5 family member HSPA13A was increased in human dystrophic muscles. Likewise, HSP90B1, a molecular chaperone involved in quality control, protein folding, calcium homeostasis, and ERAD (Eletto et al., 2010), was upregulated by dystrophin deficiency. Distinct from the D2-mdx mice, CHOP/DDIT3/GADD153 was similar in healthy and dystrophin-deficient muscle from humans. PDIA5 and PDIA3, PDI genes that enable the formation of disulfide bonds and facilitate protein folding (Kranz et al., 2017; Adams et al., 2019), were upregulated in human dystrophic muscles. PTPN2, which regulates ER stress (Kasper et al., 2015), and, in concordance with previous findings (Moorwood and Barton, 2014), CASP4 expression were upregulated in human dystrophic muscles. Resolution to discordant expression of protein from D2-mdx mice and transcripts from boys with DMD will require further inquiry, but may be driven by differences in disease progression, animal type, and/or translation, among other factors.

To better understand the regulation of the genes involved in ER stress and the UPR in dystrophic muscles, we used iRegulon, a Cytoscape plugin based on motif detection and track discovery (Janky et al., 2014). Through the cis-regulatory sequence analyses in iRegulon (Janky et al., 2014), we identified transcriptional regulators of the genes of interest. Our approach revealed a total of 55 transcriptional regulators and orthologues at NES > 3.00. Among the putative transcription factors and orthologues identified, experimental evidence demonstrates that XBP1, ATF6, and CEBP are directly involved in the upregulation of UPR genes (Afroze and Kumar, 2019). In addition, the FOXO transcription factors (FOXO1 and FOXA1) were also identified in our analysis, and are well-known to regulate skeletal muscle homeostasis through their involvement in the modulation of energy homeostasis, proteolysis pathways, apoptosis, and regeneration (Sanchez et al., 2014; Parolo et al., 2018). Furthermore, based on predicted String interactions, the transcriptional regulators, TAF9, NFYA, and TP53, have previous experimental evidence of existing interactions between these transcriptional regulators and genes. These transcriptional regulators, to our knowledge, were not previously implicated in relation to ER stress in DMD. Interestingly, NFYA was previously identified as a regulator of regeneration and tissue repair (Musarò, 2020). Since ER stress and the UPR may also play a role in other myopathies, including LGMDs and Miyoshi myopathy (Ikezoe et al., 2003; Boito et al., 2007; Bohnert et al., 2018), findings reported herein may extend to a broader array of disease states.

In total, we provide novel evidence that dystrophin deficiency causes upregulation of ER stress and UPR markers in muscle from D2-mdx mice. Parallel to this important finding, we also demonstrated that similar alterations in ER stress and the UPR occur in human dystrophic muscle at the transcript level, and we identified key transcription factors, which appear to be involved in the regulation of these processes. We found that some genes are unpaired in terms of ER stress and UPR regulation in D2-mdx mice and human dystrophic skeletal muscles, requiring further studies to clarify the pattern of these events in the proposed experimental model. While acute activation of ER stress and the UPR and subsequent stimulation of proteolytic systems are likely a means to counter cell stress, it is likely that in dystrophic muscle chronic activation of ER stress and the UPR contributes to pathology, particularly as inhibition of ER stress via ablation of caspase-12 attenuates disease severity in mdx mice (Moorwood and Barton, 2014). Likewise, the therapeutic impact of calpain and proteasome inhibition are equivocal and collectively range from supportive of and antagonistic of cell health (Spencer and Mellgren, 2002; Gazzerro et al., 2010; Selsby et al., 2010; Selsby et al., 2012). It is reasonable to suggest that the UPR, autophagy, and proteolytic systems are entangled, however, their integrated responses to acute and chronic activation underscore the complexities of these systems and make clear that there are distinctions between outcomes driven acute and chronic activation. These data provide valuable insight regarding the regulation of ER stress in dystrophic muscle and support the possibility that strategies for maintenance of ER homeostasis may be of therapeutic importance.

## Figures and Tables

**Table:** 

**AP1**	Activator protein 1
**ATF4**	Activating transcription factor 4
**ATF6**	Activating transcription factor 6
**BCA**	Bicinchoninic acid
**BiP**	Binding-immunoglobulin protein
**CEBP**	CCAAT-enhancer-binding proteins
**CHOP**	C/EBP homologous rotein
**CREB3**	Cyclic-AMP response element binding protein 3
**CREB3L1**	Cyclic-AMP response element binding protein 3L1
**CREB3L2**	Cyclic-AMP response element binding protein 3L2
**DDIT3**	DNA damage inducible transcript 3
**EIF2AK2**	Eukaryotic translation initiation factor 2 alpha kinase 2
**EIF2AK3**	Eukaryotic translation initiation factor 2 alpha kinase 3
**eIF2α**	Eukaryotic translation initiation factor 2 alpha
**ERK**	Extracellularly regulated kinases
**FOXA1**	Forkhead
**FOXO**	Forkhead box O
**FOXO1**	Forkhead box O1
**GADD153**	Growth arrest and DNA damage-inducible gene
**GCN2**	General control non-derepressible 2
**GRP78**	78 kDa glucose-regulated protein
**HRI**	Heme-regulated inhibitor
**HSP90B1**	Heat shock 70 kDa protein 90B1
**HSPA13A**	Heat shock 70 kDa protein 13 A
**HSPA5**	Heat shock 70 kDa protein 5
**IRE1α**	Inositol-requiring enzyme 1alpha
**JNK**	C-Jun N-terminal kinase
**LGMD**	Limb girdle muscular dystrophy
**NFYA**	Nuclear transcription factor Y subunit alpha
**NFκB**	Nuclear factor of kappa light polypeptide gene enhancer in B-cells
**PDIA3**	Protein disulfide isomerase family A member 6
**PDIA5**	Protein disulfide isomerase family A member 5
**PKR**	Protein kinase R
**PTPN2**	Protein tyrosine phosphatase non-receptor type 2
**SDS**	Sodium dodecyl sulfate
**TAF9**	TATA box binding protein (TBP)-associated factor 9
**TP53**	Tumor protein 53
**XBP1**	X-box binding protein 1

## Data Availability

Publicly available datasets were analyzed in this study. This data can be found here: https://www.ncbi.nlm.nih.gov/geo/query/acc.cgi?acc&equals;GSE38417. Affymetrix microarray data set accession ID: GSE38417 (alternate ID: EGEOD-38417).
