# Supplementary material for: Indicators of increased ER stress and UPR in aged D2-mdx and human dystrophic skeletal muscles
Source: Front Physiol. 2023 Apr 25;14:1152576. doi: 10.3389/fphys.2023.1152576 (PMC10166835; doi:10.3389/fphys.2023.1152576)

**Supplementary data**

Supplementary table 1. ER stress-related genes and their fold changes identified based on the sub-setting on the genes from GO biological and cellular components identified from the upregulated genes following Affymetrix chip analysis.

|  | **Genes** | **Gene names** | **Fold change** | **p-value** | **FDR** |
| --- | --- | --- | --- | --- | --- |
| 1 | ALOX5 | Arachidonate 5-lipoxygenase | 2.3036 | 6.00E-10 | 3.00E-05 |
| 2 | ARFGAP3 | ADP-ribosylation factor GTPase-activating protein 3 | 0.753 | 1.00E-06 | 0.072 |
| 3 | ATXN3 | Ataxin-3 | 1.1377 | 6.00E-10 | 3.00E-05 |
| 4 | B2M | Beta-2-microglobulin | 0.978 | 9.00E-13 | 5.00E-08 |
| 5 | BID | BH3-interacting domain death agonist | 1.5672 | 3.00E-09 | 2.00E-04 |
| 6 | CANX | Calnexin | 1.1714 | 6.00E-09 | 3.00E-04 |
| 7 | CASP4 | Caspase-4 | 1.8583 | 3.00E-13 | 2.00E-08 |
| 8 | CAV1 | Caveolin-1 | 0.8222 | 1.00E-08 | 8.00E-04 |
| 9 | CCDC47 | Coiled-coil domain-containing protein 47 | 1.4462 | 1.00E-11 | 6.00E-07 |
| 10 | CD74 | HLA class II histocompatibility antigen gamma chain | 1.1424 | 5.00E-06 | 0.261 |
| 11 | CREB3L2 | Cyclic AMP-responsive element-binding protein 3-like protein | 1.223 | 2.00E-09 | 1.00E-04 |
| 12 | CRTAP | Cartilage-associated protein | 1.0608 | 2.00E-08 | 0.001 |
| 13 | CSNK2A2 | Casein kinase II subunit alpha | 0.9034 | 2.00E-09 | 1.00E-04 |
| 14 | DNAJB11 | DnaJ homolog subfamily B member 11 | 0.7875 | 2.00E-08 | 9.00E-04 |
| 15 | DNAJB14 | DnaJ homolog subfamily B member 14 | 1.2192 | 2.00E-13 | 1.00E-08 |
| 16 | DNAJC10 | DnaJ homolog subfamily C member 10 | 0.8331 | 1.00E-08 | 6.00E-04 |
| 17 | EDEM1 | ER degradation-enhancing alpha-mannosidase-like protein 1 | 0.9162 | 1.00E-10 | 6.00E-06 |
| 18 | EDEM3 | ER degradation-enhancing alpha-mannosidase-like protein 3 | 0.9737 | 3.00E-06 | 0.123 |
| 19 | ERLIN1 | Erlin-1 | 0.964 | 1.00E-10 | 8.00E-06 |
| 20 | ERLIN2 | Erlin-2 | 0.7998 | 4.00E-10 | 2.00E-05 |
| 21 | EXTL2 | Exostosin-like 2 | 0.7699 | 3.00E-08 | 0.001 |
| 22 | FKBP14 | Peptidyl-prolyl cis-trans isomerase FKBP14 | 1.1832 | 4.00E-12 | 3.00E-07 |
| 23 | FKBP1B | Peptidyl-prolyl cis-trans isomerase FKBP1B | 1.2152 | 6.00E-09 | 3.00E-04 |
| 24 | GAK | Cyclin-G-associated kinase | 0.8136 | 1.00E-09 | 7.00E-05 |
| 25 | HSP90B1 | Endoplasmin | 0.7424 | 1.00E-06 | 0.059 |
| 26 | HSPA13 | Heat shock 70 kDa protein 13 | 1.1258 | 4.00E-09 | 2.00E-04 |
| 27 | ITPR1 | Inositol 1,4,5-trisphosphate receptor type 1 | 1.2345 | 4.00E-10 | 2.00E-05 |
| 28 | KDELR3 | ER lumen protein-retaining receptor 3 | 1.8423 | 4.00E-11 | 2.00E-06 |
| 29 | LEPRE1 | Procollagen-proline 3-dioxygenase 1 | 1.1587 | 3.00E-11 | 2.00E-06 |
| 30 | MAN1A1 | Mannosyl-oligosaccharide 1,2-alpha-mannosidase IA | 2.1804 | 6.00E-14 | 4.00E-09 |
| 31 | MAN1B1 | ER mannosyl-oligosaccharide 1,2-alpha-mannosidase | 0.5523 | 3.00E-08 | 0.002 |
| 32 | MAP3K5 | Mitogen-activated protein kinase kinase kinase 5 | 1.4522 | 1.00E-10 | 7.00E-06 |
| 33 | OS9 | Protein OS-9 | 0.8544 | 1.00E-08 | 6.00E-04 |
| 34 | PDIA3 | Protein disulfide-isomerase A3 | 1.259 | 9.00E-12 | 5.00E-07 |
| 35 | PDIA5 | Protein disulfide-isomerase A5 | 1.2082 | 9.00E-09 | 5.00E-04 |
| 36 | PIK3R1 | Phosphatidylinositol 3-kinase regulatory subunit alpha | 1.0914 | 1.00E-07 | 0.006 |
| 37 | PPIB | Peptidyl-prolyl cis-trans isomerase B | 1.4832 | 7.00E-09 | 4.00E-04 |
| 38 | PTPN2 | Tyrosine-protein phosphatase non-receptor type 2 | 0.6968 | 8.00E-07 | 0.039 |
| 39 | SEC16A | Protein transport protein Sec16A | 0.715 | 4.00E-08 | 0.002 |
| 40 | SEP15 | Selenoprotein f | 0.9125 | 2.00E-08 | 0.001 |
| 41 | SERP1 | Stress-associated endoplasmic reticulum protein 1 | 1.3937 | 1.00E-10 | 6.00E-06 |
| 42 | SERPINH1 | Serpin H1 | 1.0945 | 1.00E-07 | 0.008 |
| 43 | SRPX | Sushi repeat-containing protein SRPX | 3.3032 | 1.00E-13 | 8.00E-09 |
| 44 | SSR1 | Translocon-associated protein subunit alpha | 1.4661 | 3.00E-14 | 2.00E-09 |
| 45 | THBS1 | Thrombospondin-1 | 2.8867 | 8.00E-08 | 0.004 |
| 46 | TM7SF3 | Transmembrane 7 superfamily member 3 | 0.4957 | 4.00E-07 | 0.02 |
| 47 | TMCO1 | Calcium load-activated calcium channel | 0.5396 | 6.00E-08 | 0.003 |
| 48 | TMEM33 | Transmembrane protein 33 | 0.6651 | 2.00E-07 | 0.013 |
| 49 | TMTC3 | Transmembrane and TPR repeat-containing protein 3 | 1.4261 | 6.00E-14 | 3.00E-09 |
| 50 | TMX1 | Thioredoxin-related transmembrane protein 1 | 1.2266 | 3.00E-12 | 2.00E-07 |
| 51 | TP53 | Cellular tumor antigen p53 | 0.5876 | 2.00E-07 | 0.008 |
| 52 | TPP1 | Tripeptidyl-peptidase 1 | 0.763 | 6.00E-07 | 0.03 |
| 53 | TRIM25 | E3 ubiquitin/ISG15 ligase TRIM25 | 0.9594 | 8.00E-08 | 0.004 |
| 54 | UBE2J1 | Ubiquitin-conjugating enzyme E2 J1 | 0.9456 | 2.00E-09 | 1.00E-04 |
| 55 | UFL1 | E3 UFM1-protein ligase 1 | 0.9552 | 6.00E-07 | 0.029 |
| 56 | UFM1 | Ubiquitin-fold modifier 1 | 0.8275 | 3.00E-07 | 0.014 |
| 57 | UGGT2 | UDP-glucose:glycoprotein glucosyltransferase 2 | 0.863 | 2.00E-08 | 8.00E-04 |
| 58 | VAPA | Vesicle-associated membrane protein-associated protein A | 0.37 | 1.00E-06 | 0.074 |

Supplementary table 2. Transcriptional regulators and orthologues identified through iRegulon and their fold changes. The fold changes are obtained following differential expression of the Affymetrix chip data set. Analysis in iRegulon was performed to identify the transcriptional regulators of the 58 upregulated genes that are linked to the identified ER stress and UPR processes.

|  | **TFs** | **TF names** | **Fold change** | **FDR** | **p-value** |
| --- | --- | --- | --- | --- | --- |
| 1 | ATF4 | Activating transcription factor 4 | -0.5727 | 0.00061 | 1.11E-08 |
| 2 | ATF6 | Activating transcription factor 6 | 0.6592 | 0.99995 | 0.0000395 |
| 3 | ATF6B | Activating transcription factor 6 beta | 0.1154 | 0.99995 | 0.13567258 |
| 4 | CDX1 | Caudal type homeobox 1 | -0.1254 | 0.99995 | 0.14379702 |
| 5 | CREB3 | cAMP responsive element binding protein 3 | 0.1941 | 0.99995 | 0.034349 |
| 6 | CREB3L1 | cAMP responsive element binding protein 3 like 1 | 1.1140 | 0.11683 | 0.00000238 |
| 7 | CREB3L2 | cAMP responsive element binding protein 3 like 2 | 1.2237 | 0.00011 | 2E-09 |
| 8 | EIF2AK3 | Eukaryotic translation initiation factor 2 alpha kinase 3 | 0.1877 | 0.99995 | 0.574859 |
| 9 | ELF1 | E74 like ETS transcription factor 1 | 0.8334 | 0.00522 | 9.8E-08 |
| 10 | ELF3 | E74 like ETS transcription factor 3 | -0.7120 | 2.55E-05 | 4.44E-10 |
| 11 | ELF3 | E74 like ETS transcription factor 3 | -0.0487 | 0.99995 | 0.56373793 |
| 12 | ELF4 | E74 like ETS transcription factor 4 | 1.3701 | 4.1E-06 | 7.1E-11 |
| 13 | ELF5 | E74 like ETS transcription factor 5 | -0.0727 | 0.99995 | 0.3759865 |
| 14 | ELK1 | ETS transcription factor ELK1 | 0.1666 | 0.99995 | 0.05443648 |
| 15 | ELK3 | ETS transcription factor ELK3 | 1.9307 | 6E-12 | 1E-16 |
| 16 | ELK4 | ETS transcription factor ELK4 | 0.2076 | 0.99995 | 0.08447351 |
| 17 | ERF | ETS2 repressor factor | -0.3276 | 0.99995 | 0.02101219 |
| 18 | ERG | ETS transcription factor ERG | 0.8089 | 0.99995 | 0.0000248 |
| 19 | ETS1 | ETS proto-oncogene 1, transcription factor | 0.9714 | 1.3E-07 | 2.12E-12 |
| 20 | ETS2 | ETS proto-oncogene 2, transcription factor | 0.5219 | 0.99995 | 0.00039888 |
| 21 | ETV1 | ETS variant transcription factor 1 | 0.8339 | 0.99995 | 0.0000649 |
| 22 | ETV2 | ETS variant transcription factor 2 | -0.4799 | 0.35089 | 0.0000074 |
| 23 | ETV3 | ETS variant transcription factor 3 | 0.3420 | 0.99995 | 0.00195487 |
| 24 | ETV4 | ETS variant transcription factor 4 | 0.1910 | 0.99995 | 0.13447787 |
| 25 | ETV5 | ETS variant transcription factor 5 | 1.1062 | 0.00112 | 2.04E-08 |
| 26 | ETV6 | ETS variant transcription factor 6 | 0.7398 | 0.03075 | 6.02E-07 |
| 27 | ETV6 | ETS variant transcription factor 6 | 0.8808 | 0.99995 | 0.0000286 |
| 28 | FEV | FEV transcription factor, ETS family member | -0.1829 | 0.99995 | 0.03871829 |
| 29 | FLI1 | Fli-1 proto-oncogene, ETS transcription factor | 1.4961 | 4.8E-09 | 8.02E-14 |
| 30 | GABPA | GA binding protein transcription factor subunit alpha | 1.0043 | 0.00399 | 7.44E-08 |
| 31 | GABPB1 | GA binding protein transcription factor subunit beta 1 | 0.4926 | 0.99995 | 0.0000279 |
| 32 | HEY1 | hes related family bHLH transcription factor with YRPW motif 1 | 0.5192 | 0.99995 | 0.00492365 |
| 33 | HNF1A | HNF1 homeobox A | -0.5213 | 0.00031 | 5.46E-09 |
| 34 | HNF1A | HNF1 homeobox A | -0.1702 | 0.99995 | 0.03185594 |
| 35 | HNF1B | HNF1 homeobox B | 0.0979 | 0.99995 | 0.26003339 |
| 36 | MYB | MYB proto-oncogene, transcription factor | 0.4464 | 0.99995 | 0.00056931 |
| 37 | NFATC3 | Nuclear factor of activated T cells 3 | -0.7510 | 8.14E-06 | 1.40E-10 |
| 38 | NFATC3 | Nuclear factor of activated T cells 3 | 0.0180 | 0.99995 | 0.8535575 |
| 39 | NFE2L1 | NFE2 like bZIP transcription factor 1 | -0.2404 | 0.99995 | 0.00547528 |
| 40 | NFYA | Nuclear transcription factor Y subunit alpha | 0.6804 | 0.06662 | 0.00000133 |
| 41 | NFYB | Nuclear transcription factor Y subunit beta | -0.6365 | 0.02724 | 5.31E-07 |
| 42 | NFYB | Nuclear transcription factor Y subunit beta | 0.1279 | 0.99995 | 0.08992659 |
| 43 | NFYC | Nuclear transcription factor Y subunit gamma | -0.7175 | 0.08259 | 1.66E-06 |
| 44 | NFYC | Nuclear transcription factor Y subunit gamma | -0.3161 | 0.99995 | 0.00342897 |
| 45 | PAX4 | Paired box 4 | -0.0363 | 0.99995 | 0.60312613 |
| 46 | POLE3 | DNA polymerase epsilon 3, accessory subunit | 0.1803 | 0.99995 | 0.04855756 |
| 47 | SRRM3 | Serine/arginine repetitive matrix 3 | -0.3630 | 0.45164 | 0.00000962 |
| 48 | TAF9 | TATA-box binding protein associated factor 9 | 0.4325 | 0.99995 | 0.0000265 |
| 49 | TLX2 | T cell leukemia homeobox 2 | -1.0053 | 1.37E-05 | 2.37E-10 |
| 50 | TLX2 | T cell leukemia homeobox 2 | -0.3525 | 0.99995 | 0.00012026 |
| 51 | TP53 | Tumor protein p53 | 0.5876 | 0.008 | 1.51E-07 |
| 52 | XBP1 | X-box binding protein 1 | #N/A | #N/A | #N/A |
| 53 | ZEB1 | Zinc finger E-box binding homeobox 1 | -0.0008 | 0.99995 | 0.9958873 |
| 54 | ZEB1-AS1 | ZEB1 antisense RNA 1 | 0.8938 | 0.07422 | 1.49E-06 |
| 55 | ZNF143 | Zinc finger protein 143 | 0.2554 | 0.99995 | 0.01281881 |

Supplementary figure 1. Transcriptional regulators and genes of interest that have experimentally determined interactions based on identified interactions using String. An interaction confidence cut-off of 0.7 was used.


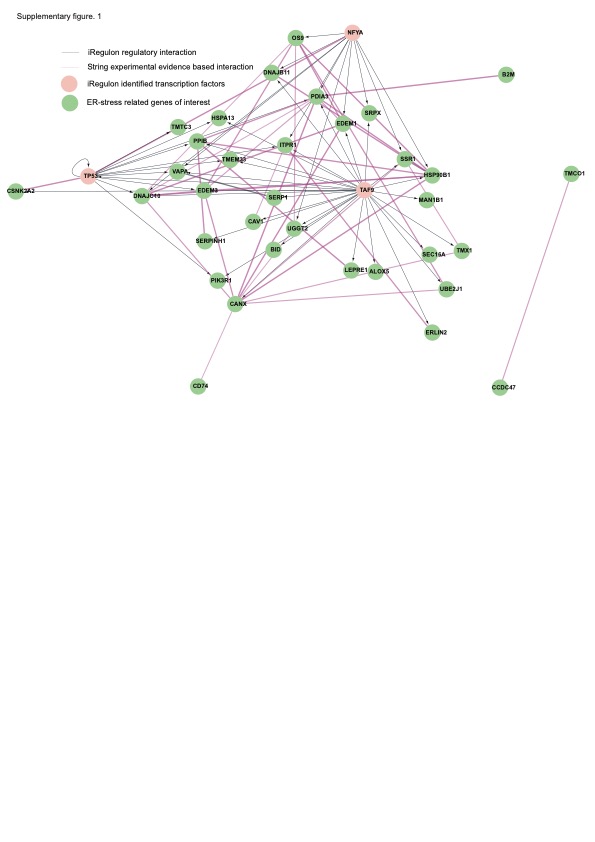

Supplement: Supplementary file 1 [file Table1.DOCX]
